# Hydrogen bonds and π–π inter­actions in two new crystalline phases of methyl­ene blue

**DOI:** 10.1107/S2056989017017881

**Published:** 2018-04-17

**Authors:** Stefano Canossa, Giovanni Predieri, Claudia Graiff

**Affiliations:** a Department of Chemistry, Life Sciences and Environmental Sustainability, University of Parma, Parco Area delle Scienze, 17/A 43124 Parma, Italy

**Keywords:** crystal structure, methyl­ene blue, chloride, bis­ulfite, hydrogen bonding, π–π inter­actions, Hirshfeld surface analysis

## Abstract

Two unprecedented solid phases involving the 3,7-bis­(di­methyl­amino)­pheno­thia­zin-5-ium cation, *i.e.* methyl­ene blue (**MB^+^**), have been obtained and structurally characterized. In the crystals of 3,7-bis­(di­methyl­amino)­pheno­thia­zin-5-ium chloride dihydrate, C_16_H_18_N_3_S^+^·Cl^−^·2H_2_O (**I**) and 3,7-bis­(di­methyl­amino)­pheno­thia­zinium bis­ulfite, C_16_H_18_N_3_S^+^·HSO_4_^−^ (**II**), the cationic dye mol­ecules are planar and disposed in an anti­parallel mode, showing π–π stacking inter­actions, with mean inter­planar distances of 3.326 (4) and 3.550 (3) Å in (**I**) and (**II**), respectively. In compound (**I**), whose phase was found affected by merohedral twinning [BASF = 0.185 (3)], the presence of water mol­ecules allows a network of hydrogen bonds involving **MB^+^** as both a donor and an acceptor, whereas in compound (**II**), the homo-inter­action of the anions causes an effective absence of classical hydrogen-bond donors. This substantial difference has important consequences for the stacking geometry and supra­molecular inter­actions of the **MB^+^** cations, which are analysed by Hirshfeld fingerprint plots and subsequently discussed.

## Abstract 2

Two unprecedented solid phases of methyl­ene blue (**MB** ^+^), *viz.* 3,7-bis­(di­methyl­amino)­pheno­thia­zin-5-ium chloride dihydrate and 3,7-bis­(di­methyl­amino)­pheno­thia­zin-5-ium bis­ulfite, have been obtained and structurally characterized. The effective absence of hydrogen-bond donors in the second compound has important consequences on the stacking geometry and supra­molecular inter­actions of the **MB^+^** ions, which are analysed by Hirshfeld fingerprint plots.

## Chemical context   

The 3,7-bis­(di­methyl­amino)­pheno­thia­zin-5-ium ion, better known as **methyl­ene blue cation** (**MB^+^**), is a renowned compound with important applications in medicine (Hanzlik, 1933 ▸; Wendel, 1935 ▸; Wischik *et al.*, 1996 ▸), biology (Jung & Metzger, 2013 ▸; Färber *et al.*, 1998 ▸) and chemistry (Bergamonti *et al.*, 2015 ▸; Kim *et al.*, 2014 ▸). **MB^+^**, with formula C_16_H_18_N_3_S^+^, consists of three condensed six-membered rings with two heteroatoms in the central one, and two terminal di­methyl­amine groups. The delocalization of the +1 charge, which involves the whole mol­ecule with the exception of the four peripheral methyl groups, causes an overall planarity and the typical intense blue colour exhibited by **MB^+^** solutions in many solvents. The formal resonant structures are shown in the Scheme.

The **MB^+^** chloride salt is the first fully synthetic drug to be used in medicine, originally as an anti­malarial agent (Coulibaly *et al.*, 2009 ▸), an anti­depressant (Eroğlu & Çağlayan, 1997 ▸), an anti­hemoglobinemic (Cawein *et al.*, 1964 ▸) and as a disinfectant (Lo *et al.*, 2014 ▸). In chemistry, it has various colourimetric and photocatalytic uses (Hang & Brindley, 1970 ▸; Kim *et al.*, 2014 ▸; Bergamonti *et al.*, 2015 ▸), which rely on its capability of undergoing a reduction process in the presence of weak reducing agents, turning into the colourless leuko­methyl­ene blue. The latter, in turn, can be oxidized to restore the original **MB^+^** cation, and this feature makes it a valid redox agent in biochemistry where it plays relevant roles in the study of enzyme-catalysed redox reactions. Recently, despite the cationic nature of **MB^+^**, we found that its peculiar electronic situation enables ligand behaviour towards *M*Cl_2_ fragments (*M* = Cu and Ag) through the central aromatic nitro­gen atom (Canossa *et al.*, 2017 ▸), thus proving that some properties of this common and widespread mol­ecule are still to be discovered.

Commercial **MB** is a penta­hydrate chloride salt, whose structure was reported in 1973 (Marr *et al.*, 1973 ▸). Recently, Rager *et al.* (2012 ▸) reinvestigated its crystalline states at variable temperatures, which led to the observation of five different hydrates with clearly distinct structures, as shown by powder X-ray diffraction analyses. However, no structural data are available and, to date, only the structure of the commercial penta­hydrate form is known. Herein, we report and discuss the mol­ecular and crystal structures of the unreported dihydrate phase of **MB^+^** chloride (**I**), one of those predicted by Rager *et al.* (2012 ▸), and the crystal structure of a new anhydrous form of MB^+^ bis­ulfite (**II**).
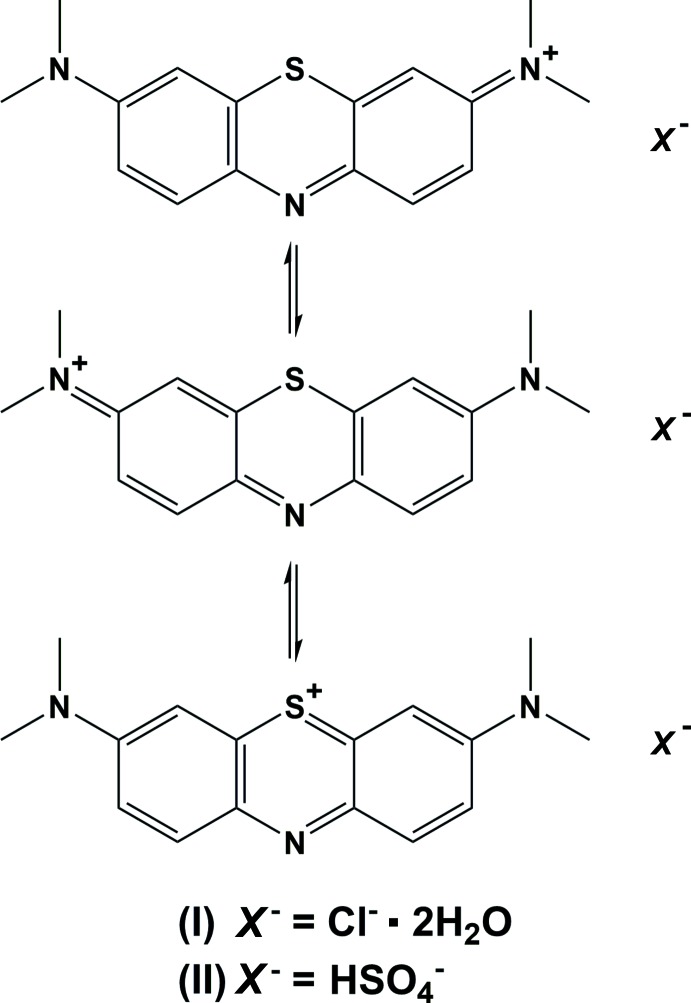


## Structural commentary   

**Figure 1:**
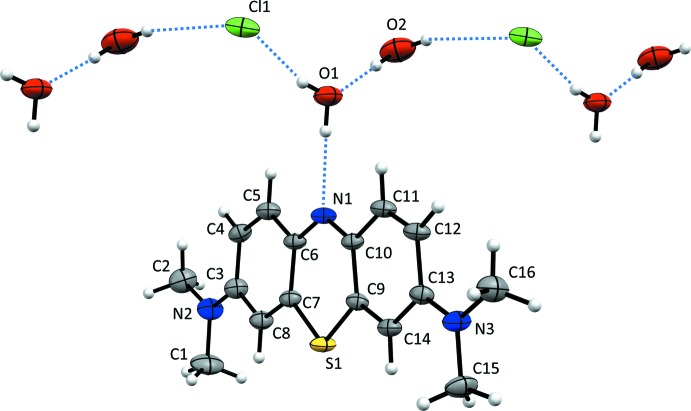
The mol­ecular structure of compound (**I**), with the atom labelling. Displacement ellipsoids are drawn at the 50% probability level. Hydrogen bonds are shown as dashed lines (see Table 1 ▸).

**Figure 2:**
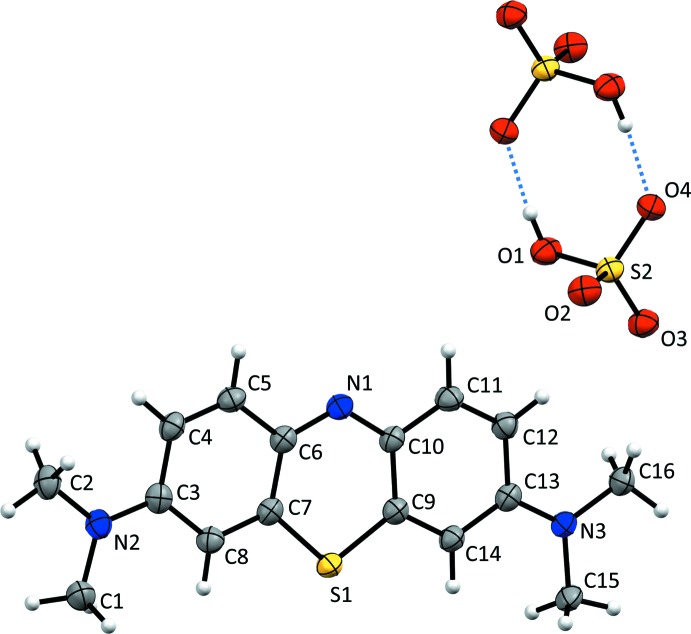
The mol­ecular structure of compound (**II**), with the atom labelling. Displacement ellipsoids are drawn at the 50% probability level. Hydrogen bonds are shown as dashed lines (see Table 2 ▸).

The mol­ecular structures of compounds (**I**) and (**II**) are illus­trated in Figs. 1 ▸ and 2 ▸, respectively. Details of the hydrogen bonding in the crystals of compounds (**I**) and (**II**) are given in Tables 1 ▸ and 2 ▸, respectively. In compound (**I**), the asymmetric unit is composed of one **MB^+^** cation, a chloride anion, and two water mol­ecules. The latter are linked head-to-tail by O—H⋯O hydrogen bonds which, in turn, are linked by O—H⋯Cl hydrogen bonds, forming chains propagating along [001], as shown in Fig. 1 ▸ (see also Table 1 ▸). The asymmetric unit of compound (**II**) consists of an **MB^+^** cation and a bis­ulfite anion. In both compounds, the **MB^+^** cations display a typical resonance structure, as evidenced by the values of the C—C bond lengths in the rings, which range from 1.352 (3) to 1.447 (5) Å. This bond-length distribution range is the same as that observed in other reported structures containing **MB^+^** cations, for example, as for its chloride penta­hydrate form (Marr *et al.*, 1973 ▸). The two C—S bond lengths, S1—C7 and S1—C9 [respectively, 1.731 (4) and 1.734 (4) Å in (**I**) and 1.732 (2) and 1.727 (2) Å in (**II**)], are very similar and in agreement with analogous data reported in the literature. The **MB^+^** cations are planar considering the three condensed six-membered rings [atoms S1/N1/C3–C14; r.m.s. deviations are 0.011 Å for (**I**) and 0.01 Å for (**II**)] and the external di­methyl­amine groups, with the only exception being the aliphatic hydrogen atoms. In compound (**II**), one of the four S—O bond lengths of the bis­ulfite anion [S2—O1 = 1.575 (3) Å] is longer than the other three, which vary from 1.439 (2) to 1.468 (2) Å, thus confirming the identity of the OH group in this anion. The anions are linked by a pair of O—H⋯O hydrogen bonds forming an inversion dimer (Fig. 2 ▸ and Table 2 ▸).

## Supra­molecular features   

**Figure 3:**
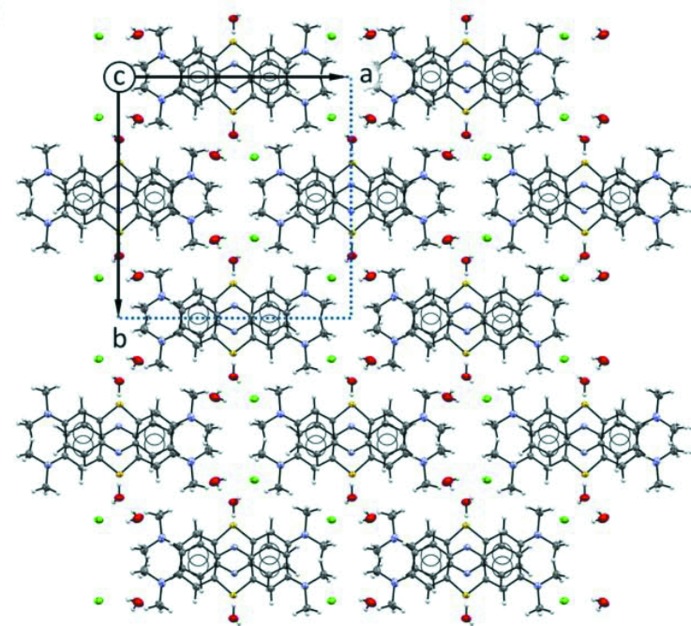
The crystal packing of compound (**I**) viewed along the *c* axis, with the unit cell highlighted in the upper left-hand corner. Displacement ellipsoids are drawn at the 50% probability level.

**Figure 5:**
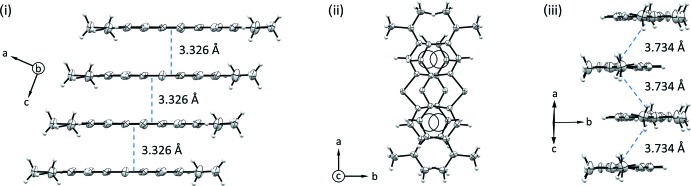
Views of the stacking geometry of **MB^+^** in compound (**I**): (i) displayed orthogonally to the stacking pillar axis by showing a tetra­mer of stacked mol­ecules; (ii) the same group of **MB^+^** cations is shown along the stacking direction; (iii) view along the **MB^+^** longer dimension, highlighting the mutual shifts of the cations in the zigzag columns.

**Figure 6:**
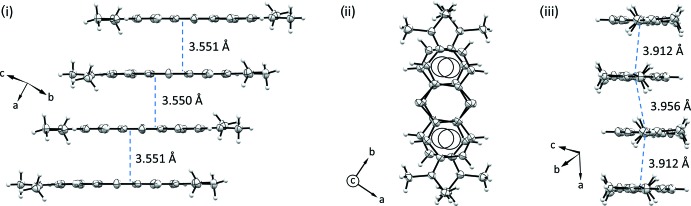
Views of the stacking geometry of **MB^+^** in compound (**II**): (i) displayed orthogonally to the stacking pillar axis by showing a tetra­mer of stacked mol­ecules; (ii) the same group of **MB^+^** cations is shown along the stacking direction; (iii) view along the **MB^+^** longer dimension, highlighting the nearly completely eclipsed superposition of the cations in the anti­parallel columns.

In the crystal packing of the two compounds, illustrated in Figs. 3 ▸ and 4 ▸, the planar **MB^+^** cations are stacked in an anti­parallel mode, with the sulfur atom disposed alternatively on opposite sides. The aromatic systems exhibit offset π–π inter­actions and form infinite layers as shown in Figs. 5 ▸ and 6 ▸. The average inter­planar distances are 3.326 (4) Å in (**I**) and 3.550 (3) Å in (**II**). This disposition differs from the one observed in the penta­hydrate form where the **MB^+^** species are stacked together while adopting the same orientation, so that the sulfur atoms of all of the mol­ecules lie on the same side along the stacking column. Moreover, as evidenced in Fig. 5 ▸ii–iii and Fig. 6 ▸ii–iii, the stacking geometry of **MB^+^** differs significantly in the two phases. In fact, in the case of (**I**), the anti­parallel mode is accompanied by a mutual shift of the cations, resulting in the formation of a zigzag chain with an inter-centroid distance between central thia­zine rings of 3.734 (3) Å (Fig. 5 ▸iii). On the other hand, in (**II**) the stacked mol­ecules are almost eclipsed and the equivalent inter-centroid distances are 3.912 (4) and 3.956 (5) Å (Fig. 6 ▸iii).

## Hirshfeld surface analysis   

**Figure 7:**
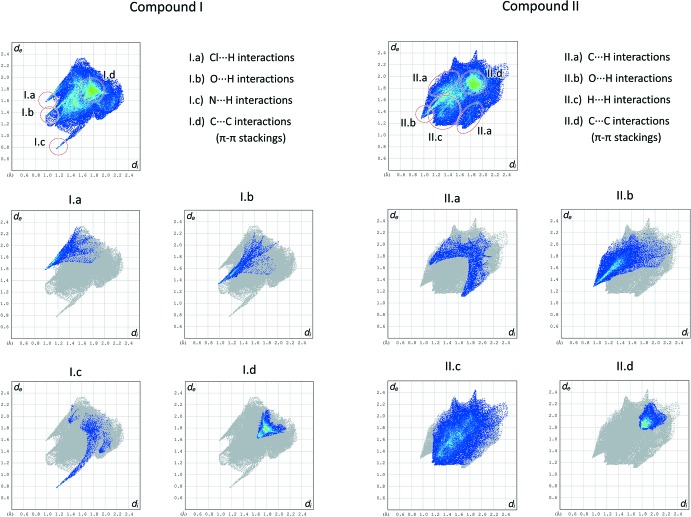
Hirshfeld fingerprint plots of compounds (**I**) and (**II**) (above) and some relevant components (I.a–d and II.a–d), highlighting the main inter­actions exhibited by **MB^+^** in the respective solid phases.

An evaluation of the Hirshfeld fingerprint plots (Spackman & Jayatilaka, 2009 ▸) of compounds (**I**) and (**II**), shown in Fig. 7 ▸, highlights some differences in the inter­actions of the **MB^+^** cations in the two phases. In phase (**I**), the leading inter­actions can be grouped in two classes: hydrogen bonds and π–π stacking. The first involves **MB^+^** as a donor by means of aromatic and aliphatic C—H bonds (Table 1 ▸), and as an acceptor by means of the central N atom, whose σ lone pair pointing out of the mol­ecule is readily exploited by a water mol­ecule to form a strong hydrogen bond [N⋯O distance = 2.936 (4) Å; see Fig. 1 ▸ and Table 1 ▸). The presence of hydrogen-bond donors surrounding **MB^+^**, *i.e*. water mol­ecules, is therefore able to satisfy the region of the cation with the most prominent partial negative charge (the nitro­gen atom).

On the other hand, considering the fingerprint plot of compound (**II**), it can be seen that the strongest inter­actions are π–π stacking and C—H⋯O contacts (Table 2 ▸) between **MB^+^** and the oxygen atoms of the bis­ulfite inversion dimer. Since no available hydrogen-bond donor is present near **MB^+^**, no inter­action is able to exploit the electron density concentrated on the central N atom. This has important consequences, since, on one side, it allows a better alignment of the **MB^+^** cations in their stacking arrangement, as clearly shown in Fig. 6 ▸. However, although there is an improved geometrical match, the stacking distance increases as a consequence of the charge repulsion between the mono-cationic mol­ecules.

This evidence constitutes an exception to a general trend in the packing preferences of organic species. Indeed, in cases where both hydrogen bonds and π stacking can be found in the solid phase, the two inter­actions compete to maximize their efficiency. This competition is usually in favour of the more directional supra­molecular inter­actions, *i.e.* hydrogen bonds (Gospodinova & Tomšík, 2015 ▸). In the present case, however, the cationic nature of the aromatic mol­ecules does not favour the stacking disposition that is usually better (in energetic terms), and in the case where there are no strong hydrogen bonds involving **MB^+^**, as in compound (**II**), the mol­ecule is able to adopt a theoretically more stabilizing stacking geometry, which in this case is a less stabilizing one.

## Database survey   

In the Cambridge Structural Database (CSD, version 5.38, last updated May 2017; Groom *et al.*, 2016 ▸) the crystal structure of the 3,7-bis­(di­methyl­amino)­pheno­thia­zin-5-ium hydrogen sulfate dihydrate can be found as a Private Communication (XUVROW; Lynch, 2009 ▸). Here, it was not possible to locate the H atom of the inorganic moiety, nor those of the water mol­ecules, because of the poor data quality due to problematic twinning affecting the solid phase. Considering the overall crystal packing of this phase, which features **MB^+^** as both a hydrogen-bond donor and acceptor towards the water mol­ecules and the anion, the inter­actions of the organic cation are much more similar to those observed for compound (**I**), than those observed for compound (**II**).

A search of the CSD found 30 compounds containing the aromatic unit 3,7-bis(dimethylamino)phenothiazin-5-ium cation. The anions present in the crystal structures include inorganic halogenide, nitrate, perchlorate, thio­cyanate, tri­iodide, hydrogen sulfate and different metallates. The geometrical parameters of the cations (bond lengths, bond angles and torsion angles) are in the normal range for condensed ring systems.

## Synthesis and crystallization   

**Preparation of compound (I)**

For the crystallization of compound (**I)**, the commercial reagent 3,7-bis(dimethylamino)phenothiazin-5-ium chloride was used without any preparative treatment. 50 mg of 3,7-bis(dimethylamino)phenothiazin-5-ium chloride penta­hydrate (0.156 mmol) were transferred to a 10 ml glass vial containing 5 ml of di­chloro­methane. The container was then closed and placed in an ultrasound bath for 5 min. to reach the saturation limit of the compound. The mixture thus obtained was filtered into another 5 ml glass vial, and the resulting solution was left partially open for slow evaporation of the solvent. After 24 h, metallic dark-green needle-shaped crystals of compound (**I**), suitable for X-ray diffraction analysis, were obtained.

**Preparation of compound (II)**

306 mg of 3,7-bis(dimethylamino)phenothiazin-5-ium chloride penta­hydrate (0.957 mmol) were transferred to an agate mortar, together with 284 mg of HgSO_4_ (0.957 mmol). The two powders were subsequently mixed and ground for 30 min, resulting in a dark-green powder. X-ray powder diffraction analysis was performed on the as-obtained product. The resulting pattern showed peaks clearly belonging to the final compound (**II**) (see Fig. S1 in the supporting information). An excess of the powder was then placed in a glass vial, together with 3 ml of *N*,*N*-di­methyl­formamide. The container was closed and placed in an ultrasound bath for 5 min. to reach the saturation limit of the compound. The mixture obtained was filtered into another 5 ml glass vial, and the resulting solution was left partially open for slow evaporation of the solvent. After one week, metallic dark-green needle-like crystals of compound (**II**), suitable for X-ray diffraction analyses, were obtained.

## Refinement details   

Crystal data, data collection and structure refinement details are summarized in Table 3 ▸. For both compounds, the H atoms were positioned geometrically and refined using a riding model: C—H = 0.99 Å with *U*_iso_(H) = 1.2*U*_eq_(C). The H atoms of the water mol­ecules in (**I**) and the bis­ulfite anion in (**II**) were located in difference-Fourier maps and refined freely. Compound (**I**) was refined as a merohedral twin with twin matrix, 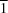 0 0, 0 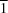 0, 0 0 1, with a refined BASF value of 0.185 (3).

Diffraction data for compound (**I**) were collected using a Bruker D8 Venture diffractometer, equipped with a CMOS PhotonII detector, a Mo High brilliance microsource (Incoatec) working at 50 KV and 1 mA. For compound (**II**), the data were collected at the ELETTRA Synchrotron facility (CNR Trieste) using monochromated 0.7 Å wavelength radiation and a Pilatus 2M Detector (Dectris).

## Supplementary Material

Crystal structure: contains datablock(s) I, II, global. DOI: 10.1107/S2056989017017881/su5412sup1.cif

Structure factors: contains datablock(s) I. DOI: 10.1107/S2056989017017881/su5412Isup2.hkl

Structure factors: contains datablock(s) II. DOI: 10.1107/S2056989017017881/su5412IIsup3.hkl

Supporting information file. DOI: 10.1107/S2056989017017881/su5412Isup4.cml

Fig. S1. Stacked XRPD patterns. DOI: 10.1107/S2056989017017881/su5412sup5.pdf

CCDC references: 1811677, 1811678

Additional supporting information:  crystallographic information; 3D view; checkCIF report

## Figures and Tables

**Figure 4:**
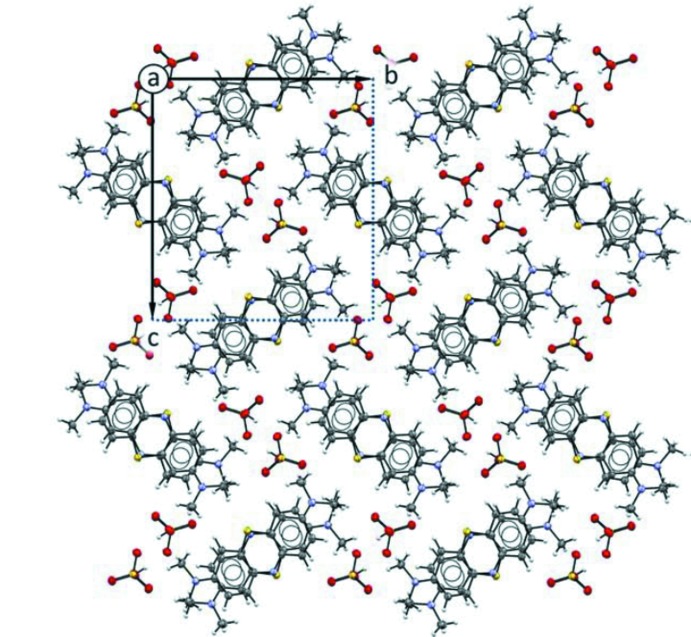
The crystal packing of compound (**II**) viewed along the *a* axis, with the unit cell highlighted in the upper left-hand corner. Displacement ellipsoids are drawn at the 50% probability level.

**Table 1:** Hydrogen-bond geometry (Å, °) for (**I**)

*D*—H⋯*A*	*D*—H	H⋯*A*	*D*⋯*A*	*D*—H⋯*A*
O1—H1*D*⋯..Cl1	0.87	2.30	3.153 (4)	168
O1—H1*E*⋯..N1	0.87	2.07	2.936 (4)	177
O2—H2*D*⋯..O1	0.87	1.97	2.837 (6)	174
O2—H2*E*⋯..Cl1^i^	0.87	2.71	3.559 (5)	165
C1—H1*B*⋯..O1^ii^	0.98	2.45	3.426 (6)	173
C2—H2*B*⋯..Cl1^iii^	0.98	2.72	3.611 (5)	152
C8—H8⋯..Cl1^iv^	0.95	2.71	3.573 (4)	152
C15—H15*B*⋯..O1^v^	0.98	2.44	3.387 (7)	162
C16—H16*A*⋯..O2^vi^	0.98	2.57	3.454 (7)	151

Symmetry codes: (i) 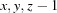; (ii) 
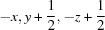
; (iii) 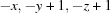; (iv) 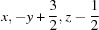; (v) 
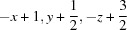
; (vi) 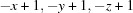.

**Table 2:** Hydrogen-bond geometry (Å, °) for (**II**)

*D*—H⋯*A*	*D*—H	H⋯*A*	*D*⋯*A*	*D*—H⋯*A*
O1—H1⋯O3^i^	0.84	1.77	2.609 (4)	175
C1—H1*C*⋯O4^ii^	0.98	2.39	3.349 (5)	167
C2—H2*B*⋯O2^iii^	0.98	2.56	3.506 (5)	163
C4—H4⋯O3^iv^	0.95	2.54	3.451 (5)	162
C12—H12⋯O4	0.95	2.46	3.372 (5)	162
C15—H15*B*⋯O2^v^	0.98	2.47	3.382 (5)	155
C15—H15*C*⋯O3^vi^	0.98	2.36	3.309 (5)	164
C16—H16*B*⋯O2^v^	0.98	2.32	3.283 (5)	167
C16—H16*C*⋯O4	0.98	2.52	3.326 (5)	139

Symmetry codes: (i) 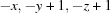; (ii) 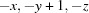; (iii) 
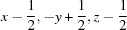
; (iv) 
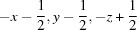
; (v) 
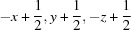
; (vi) 
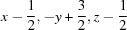
.

**Table 3:** Experimental details

	(**I**)	(**II**)
Crystal data		
Chemical formula	C_16_H_18_N_3_S^+^·Cl^−^·2H_2_O	C_16_H_18_N_3_S^+^·HSO_4_^−^
*M*_r_	355.87	381.46
Crystal system, space group	Monoclinic, *P*2_1_/*c*	Monoclinic, *P*2_1_/*n*
Temperature (K)	200	100
*a*, *b*, *c* (Å)	15.130 (2), 15.7219 (19), 7.1203 (12)	7.867 (10), 14.101 (10), 15.027 (10)
β (°)	90.600 (8)	90.348 (10)
*V* (Å^3^)	1693.6 (4)	1667 (3)
*Z*	4	4
Radiation type	Mo *K*α	Synchrotron, λ = 0.700 Å
μ (mm^−1^)	0.36	0.35
Crystal size (mm)	0.4 × 0.2 × 0.15	0.3 × 0.15 × 0.1

Data collection		
Diffractometer	Bruker D8 Venture	ELETTRA XRD1
Absorption correction	Multi-scan (*SADABS*; Krause *et al.*, 2015 ▸)	Multi-scan (*CrysAlis PRO*; Agilent, 2014 ▸)
*T*_min_, *T*_max_	0.491, 0.746	0.711, 1.000
No. of measured, independent and observed [*I* > 2σ(*I*)] reflections	12731, 3359, 2650	20743, 3367, 2795
*R*_int_	0.070	0.062
(sin θ/λ)_max_ (Å^−1^)	0.625	0.625

Refinement		
*R*[*F* ^2^ > 2σ(*F* ^2^)], *wR*(*F* ^2^), *S*	0.088, 0.271, 1.08	0.041, 0.112, 1.06
No. of reflections	3359	3367
No. of parameters	220	232
H-atom treatment	H-atom parameters constrained	H-atom parameters constrained
Δρ_max_, Δρ_min_ (e Å^−3^)	0.69, −0.62	0.36, −0.46

Computer programs: *APEX3* and *SAINT* (Bruker, 2015 ▸), *CrysAlis PRO* (Agilent, 2014 ▸), *SHELXT* (Sheldrick, 2015*a* ▸), *SHELXL2014* (Sheldrick, 2015*b* ▸), *OLEX2* (Dolomanov *et al.*, 2009 ▸) and *publCIF* (Westrip, 2010 ▸).

## supplementary crystallographic information

### 3,7-Bis(dimethylamino)-phenothiazin-5-ium chloride dihydrate (I) Crystal data

**Table:**

C_16_H_18_N_3_S^+^·Cl^−^·2H_2_O	*F*(000) = 752
*M**_r_* = 355.87	*D*_x_ = 1.396 Mg m^−^^3^
Monoclinic, *P*2_1_/*c*	Mo *K*α radiation, λ = 0.71073 Å
*a* = 15.130 (2) Å	Cell parameters from 4183 reflections
*b* = 15.7219 (19) Å	θ = 2.9–30.5°
*c* = 7.1203 (12) Å	µ = 0.36 mm^−^^1^
β = 90.600 (8)°	*T* = 200 K
*V* = 1693.6 (4) Å^3^	Needle, metallic dark green
*Z* = 4	0.4 × 0.2 × 0.15 mm

### 3,7-Bis(dimethylamino)-phenothiazin-5-ium chloride dihydrate (I) Data collection

**Table:**

Bruker D8 Venture diffractometer	2650 reflections with *I* > 2σ(*I*)
φ and ω scans	*R*_int_ = 0.070
Absorption correction: multi-scan (SADABS; Krause *et al.*, 2015)	θ_max_ = 26.4°, θ_min_ = 2.6°
*T*_min_ = 0.491, *T*_max_ = 0.746	*h* = −18→16
12731 measured reflections	*k* = −19→19
3359 independent reflections	*l* = −6→8

### 3,7-Bis(dimethylamino)-phenothiazin-5-ium chloride dihydrate (I) Refinement

**Table:**

Refinement on *F*^2^	Secondary atom site location: difference Fourier map
Least-squares matrix: full	Hydrogen site location: mixed
*R*[*F*^2^ > 2σ(*F*^2^)] = 0.088	H-atom parameters constrained
*wR*(*F*^2^) = 0.271	*w* = 1/[σ^2^(*F*_o_^2^) + (0.2*P*)^2^] where *P* = (*F*_o_^2^ + 2*F*_c_^2^)/3
*S* = 1.08	(Δ/σ)_max_ < 0.001
3359 reflections	Δρ_max_ = 0.69 e Å^−^^3^
220 parameters	Δρ_min_ = −0.62 e Å^−^^3^
0 restraints	Extinction correction: SHELXL2014 (Sheldrick, 2015b), Fc^*^=kFc[1+0.001xFc^2^λ^3^/sin(2θ)]^-1/4^
Primary atom site location: dual	Extinction coefficient: 0.068 (12)

### 3,7-Bis(dimethylamino)-phenothiazin-5-ium chloride dihydrate (I) Special details

**Table:**

Geometry. All esds (except the esd in the dihedral angle between two l.s. planes) are estimated using the full covariance matrix. The cell esds are taken into account individually in the estimation of esds in distances, angles and torsion angles; correlations between esds in cell parameters are only used when they are defined by crystal symmetry. An approximate (isotropic) treatment of cell esds is used for estimating esds involving l.s. planes.
Refinement. Refined as a 2-component twin.

### 3,7-Bis(dimethylamino)-phenothiazin-5-ium chloride dihydrate (I) Fractional atomic coordinates and isotropic or equivalent isotropic displacement parameters (Å^2^)

**Table:**

	*x*	*y*	*z*	*U*_iso_*/*U*_eq_	
S1	0.25577 (6)	0.89471 (7)	0.49946 (15)	0.0277 (4)	
Cl1	0.16600 (9)	0.41765 (7)	0.8231 (2)	0.0420 (5)	
O1	0.2548 (2)	0.5092 (2)	0.4792 (5)	0.0415 (10)	
H1D	0.2311	0.4912	0.5825	0.062*	
H1E	0.2537	0.5644	0.4890	0.062*	
N1	0.25085 (17)	0.6957 (2)	0.4980 (4)	0.0250 (9)	
N2	−0.0525 (2)	0.8556 (2)	0.2382 (6)	0.0307 (9)	
N3	0.5627 (2)	0.8421 (3)	0.7571 (7)	0.0357 (10)	
C9	0.3375 (3)	0.8241 (3)	0.5706 (6)	0.0229 (9)	
C7	0.1704 (3)	0.8285 (2)	0.4280 (6)	0.0220 (9)	
O2	0.3352 (3)	0.4241 (3)	0.1739 (8)	0.0599 (12)	
H2D	0.3141	0.4524	0.2684	0.090*	
H2E	0.2925	0.4118	0.0957	0.090*	
C10	0.3252 (3)	0.7342 (2)	0.5600 (6)	0.0229 (9)	
C1	−0.0600 (3)	0.9479 (3)	0.2297 (9)	0.0384 (13)	
H1A	−0.0629	0.9709	0.3574	0.058*	
H1B	−0.1138	0.9635	0.1599	0.058*	
H1C	−0.0084	0.9715	0.1660	0.058*	
C4	0.0280 (3)	0.7263 (3)	0.3139 (7)	0.0262 (10)	
H4	−0.0203	0.6916	0.2760	0.031*	
C3	0.0207 (3)	0.8180 (3)	0.3033 (7)	0.0244 (9)	
C12	0.4750 (3)	0.7173 (3)	0.6848 (7)	0.0277 (10)	
H12	0.5215	0.6806	0.7242	0.033*	
C6	0.1793 (3)	0.7374 (2)	0.4382 (6)	0.0220 (9)	
C5	0.1037 (3)	0.6901 (3)	0.3777 (7)	0.0263 (10)	
H5	0.1066	0.6298	0.3825	0.032*	
C8	0.0943 (3)	0.8667 (2)	0.3643 (7)	0.0254 (9)	
H8	0.0910	0.9270	0.3610	0.030*	
C13	0.4866 (3)	0.8077 (3)	0.6945 (7)	0.0291 (10)	
C11	0.3974 (3)	0.6836 (3)	0.6189 (7)	0.0274 (10)	
H11	0.3917	0.6235	0.6126	0.033*	
C14	0.4150 (3)	0.8592 (3)	0.6362 (7)	0.0288 (10)	
H14	0.4205	0.9193	0.6425	0.035*	
C15	0.5720 (4)	0.9346 (3)	0.7645 (10)	0.0494 (16)	
H15A	0.5670	0.9580	0.6373	0.074*	
H15B	0.6299	0.9492	0.8184	0.074*	
H15C	0.5253	0.9586	0.8429	0.074*	
C16	0.6382 (3)	0.7941 (3)	0.8289 (9)	0.0425 (13)	
H16A	0.6225	0.7338	0.8378	0.064*	
H16B	0.6548	0.8155	0.9537	0.064*	
H16C	0.6881	0.8008	0.7436	0.064*	
C2	−0.1328 (3)	0.8098 (3)	0.1796 (9)	0.0397 (13)	
H2A	−0.1784	0.8170	0.2750	0.060*	
H2B	−0.1193	0.7492	0.1653	0.060*	
H2C	−0.1542	0.8326	0.0595	0.060*	

### 3,7-Bis(dimethylamino)-phenothiazin-5-ium chloride dihydrate (I) Atomic displacement parameters (Å^2^)

**Table:**

	*U*^11^	*U*^22^	*U*^33^	*U*^12^	*U*^13^	*U*^23^
S1	0.0228 (6)	0.0139 (6)	0.0464 (8)	−0.0017 (3)	−0.0042 (6)	−0.0009 (4)
Cl1	0.0391 (7)	0.0225 (6)	0.0646 (10)	−0.0042 (5)	0.0082 (7)	−0.0071 (5)
O1	0.044 (2)	0.0234 (18)	0.057 (2)	0.0012 (12)	−0.005 (2)	0.0011 (13)
N1	0.0219 (18)	0.0151 (18)	0.038 (2)	−0.0011 (10)	0.005 (2)	−0.0001 (12)
N2	0.0236 (18)	0.0213 (18)	0.047 (2)	−0.0005 (14)	−0.0015 (17)	0.0009 (16)
N3	0.0221 (18)	0.034 (2)	0.051 (3)	−0.0055 (15)	−0.0016 (18)	0.0002 (18)
C9	0.0204 (18)	0.0196 (18)	0.029 (2)	−0.0017 (15)	0.0044 (17)	0.0013 (16)
C7	0.0183 (18)	0.0185 (18)	0.029 (2)	−0.0010 (14)	0.0030 (17)	−0.0015 (16)
O2	0.058 (2)	0.039 (2)	0.082 (3)	0.0142 (19)	0.014 (3)	0.006 (2)
C10	0.0212 (19)	0.0171 (18)	0.030 (2)	−0.0007 (15)	0.0068 (18)	0.0005 (16)
C1	0.026 (2)	0.025 (2)	0.064 (4)	0.0051 (17)	−0.006 (2)	0.006 (2)
C4	0.0240 (19)	0.0167 (19)	0.038 (3)	−0.0024 (15)	0.005 (2)	−0.0019 (18)
C3	0.0210 (19)	0.0211 (19)	0.031 (2)	0.0005 (15)	0.0037 (18)	−0.0020 (18)
C12	0.022 (2)	0.025 (2)	0.035 (3)	0.0034 (16)	0.004 (2)	0.0041 (19)
C6	0.0207 (18)	0.0175 (18)	0.028 (2)	−0.0010 (15)	0.0041 (17)	0.0015 (16)
C5	0.025 (2)	0.0163 (17)	0.037 (3)	−0.0016 (15)	0.004 (2)	−0.0019 (17)
C8	0.028 (2)	0.0164 (18)	0.032 (2)	−0.0002 (16)	0.0033 (19)	−0.0002 (17)
C13	0.021 (2)	0.028 (2)	0.038 (3)	−0.0015 (16)	0.002 (2)	0.001 (2)
C11	0.025 (2)	0.0196 (17)	0.038 (3)	0.0005 (16)	0.004 (2)	−0.0001 (18)
C14	0.025 (2)	0.024 (2)	0.037 (3)	−0.0034 (16)	0.002 (2)	0.0004 (19)
C15	0.033 (2)	0.031 (2)	0.085 (5)	−0.010 (2)	−0.007 (3)	−0.005 (3)
C16	0.024 (2)	0.044 (3)	0.059 (4)	−0.0019 (19)	−0.001 (2)	0.005 (3)
C2	0.024 (2)	0.033 (2)	0.062 (4)	0.0003 (18)	−0.006 (2)	−0.002 (2)

### 3,7-Bis(dimethylamino)-phenothiazin-5-ium chloride dihydrate (I) Geometric parameters (Å, º)

**Table:**

S1—C9	1.734 (4)	C9—C14	1.373 (6)
S1—C7	1.731 (4)	C7—C6	1.441 (5)
N1—C10	1.347 (5)	C7—C8	1.371 (6)
N1—C6	1.331 (5)	C10—C11	1.411 (6)
N2—C1	1.458 (6)	C4—C3	1.447 (5)
N2—C3	1.334 (6)	C4—C5	1.353 (6)
N2—C2	1.468 (6)	C3—C8	1.417 (6)
N3—C13	1.344 (6)	C12—C13	1.433 (6)
N3—C15	1.462 (6)	C12—C11	1.367 (6)
N3—C16	1.458 (6)	C6—C5	1.427 (6)
C9—C10	1.427 (5)	C13—C14	1.411 (6)
			
C7—S1—C9	103.2 (2)	C11—C10—C9	116.2 (4)
C6—N1—C10	123.9 (4)	C5—C4—C3	120.1 (4)
C1—N2—C2	114.4 (4)	N2—C3—C4	121.5 (4)
C3—N2—C1	121.3 (4)	N2—C3—C8	121.0 (4)
C3—N2—C2	124.2 (4)	C8—C3—C4	117.5 (4)
C13—N3—C15	119.6 (4)	C11—C12—C13	120.3 (4)
C13—N3—C16	125.0 (4)	N1—C6—C7	125.5 (4)
C16—N3—C15	115.3 (4)	N1—C6—C5	119.1 (4)
C10—C9—S1	121.8 (3)	C5—C6—C7	115.4 (4)
C14—C9—S1	116.5 (3)	C4—C5—C6	123.7 (4)
C14—C9—C10	121.8 (4)	C7—C8—C3	121.3 (3)
C6—C7—S1	120.9 (3)	N3—C13—C12	121.3 (4)
C8—C7—S1	117.1 (3)	N3—C13—C14	121.3 (4)
C8—C7—C6	122.0 (4)	C14—C13—C12	117.5 (4)
N1—C10—C9	124.7 (4)	C12—C11—C10	122.9 (4)
N1—C10—C11	119.1 (4)	C9—C14—C13	121.3 (4)

### 3,7-Bis(dimethylamino)-phenothiazin-5-ium chloride dihydrate (I) Hydrogen-bond geometry (Å, º)

**Table:**

*D*—H···*A*	*D*—H	H···*A*	*D*···*A*	*D*—H···*A*
O1—H1*D*···..Cl1	0.87	2.30	3.153 (4)	168
O1—H1*E*···..N1	0.87	2.07	2.936 (4)	177
O2—H2*D*···..O1	0.87	1.97	2.837 (6)	174
O2—H2*E*···..Cl1^i^	0.87	2.71	3.559 (5)	165
C1—H1*B*···..O1^ii^	0.98	2.45	3.426 (6)	173
C2—H2*B*···..Cl1^iii^	0.98	2.72	3.611 (5)	152
C8—H8···..Cl1^iv^	0.95	2.71	3.573 (4)	152
C15—H15*B*···..O1^v^	0.98	2.44	3.387 (7)	162
C16—H16*A*···..O2^vi^	0.98	2.57	3.454 (7)	151

Symmetry codes: (i) *x*, *y*, *z*−1; (ii) −*x*, *y*+1/2, −*z*+1/2; (iii) −*x*, −*y*+1, −*z*+1; (iv) *x*, −*y*+3/2, *z*−1/2; (v) −*x*+1, *y*+1/2, −*z*+3/2; (vi) −*x*+1, −*y*+1, −*z*+1.

### 3,7-Bis(dimethylamino)phenothiazinium bisulfite (II) Crystal data

**Table:**

C_16_H_18_N_3_S^+^·HSO_4_^−^	*F*(000) = 800
*M**_r_* = 381.46	*D*_x_ = 1.520 Mg m^−^^3^
Monoclinic, *P*2_1_/*n*	Synchrotron radiation, λ = 0.700 Å
*a* = 7.867 (10) Å	Cell parameters from 1235 reflections
*b* = 14.101 (10) Å	θ = 3.1–30.2°
*c* = 15.027 (10) Å	µ = 0.35 mm^−^^1^
β = 90.348 (10)°	*T* = 100 K
*V* = 1667 (3) Å^3^	Needle, metallic green
*Z* = 4	0.3 × 0.15 × 0.1 mm

### 3,7-Bis(dimethylamino)phenothiazinium bisulfite (II) Data collection

**Table:**

ELETTRA XRD1 diffractometer	2795 reflections with *I* > 2σ(*I*)
Radiation source: Elettra Synchrotron - XRD1 BL	*R*_int_ = 0.062
Rotation around the Phi axis scans	θ_max_ = 25.9°, θ_min_ = 2.0°
Absorption correction: multi-scan (CrysAlis PRO; Agilent, 2014)	*h* = −9→9
*T*_min_ = 0.711, *T*_max_ = 1.000	*k* = −17→17
20743 measured reflections	*l* = −18→18
3367 independent reflections	

### 3,7-Bis(dimethylamino)phenothiazinium bisulfite (II) Refinement

**Table:**

Refinement on *F*^2^	Secondary atom site location: difference Fourier map
Least-squares matrix: full	Hydrogen site location: inferred from neighbouring sites
*R*[*F*^2^ > 2σ(*F*^2^)] = 0.041	H-atom parameters constrained
*wR*(*F*^2^) = 0.112	*w* = 1/[σ^2^(*F*_o_^2^) + (0.0656*P*)^2^ + 0.4958*P*] where *P* = (*F*_o_^2^ + 2*F*_c_^2^)/3
*S* = 1.06	(Δ/σ)_max_ = 0.001
3367 reflections	Δρ_max_ = 0.36 e Å^−^^3^
232 parameters	Δρ_min_ = −0.46 e Å^−^^3^
0 restraints	Extinction correction: SHELXL2014 (Sheldrick, 2015b), Fc^*^=kFc[1+0.001xFc^2^λ^3^/sin(2θ)]^-1/4^
Primary atom site location: dual	Extinction coefficient: 0.0109 (15)

### 3,7-Bis(dimethylamino)phenothiazinium bisulfite (II) Special details

**Table:**

Geometry. All esds (except the esd in the dihedral angle between two l.s. planes) are estimated using the full covariance matrix. The cell esds are taken into account individually in the estimation of esds in distances, angles and torsion angles; correlations between esds in cell parameters are only used when they are defined by crystal symmetry. An approximate (isotropic) treatment of cell esds is used for estimating esds involving l.s. planes.

### 3,7-Bis(dimethylamino)phenothiazinium bisulfite (II) Fractional atomic coordinates and isotropic or equivalent isotropic displacement parameters (Å^2^)

**Table:**

	*x*	*y*	*z*	*U*_iso_*/*U*_eq_	
S2	0.11198 (6)	0.58607 (3)	0.40576 (3)	0.02746 (16)	
S1	−0.23619 (7)	0.56658 (4)	−0.09373 (3)	0.02953 (16)	
O1	−0.08785 (19)	0.57885 (11)	0.40548 (11)	0.0357 (4)	
H1	−0.1178	0.5308	0.4345	0.054*	
O3	0.16539 (19)	0.56866 (11)	0.49810 (10)	0.0336 (4)	
O2	0.1747 (2)	0.51512 (10)	0.34552 (10)	0.0359 (4)	
O4	0.1447 (2)	0.68213 (10)	0.37808 (10)	0.0349 (4)	
N3	0.0335 (2)	0.80042 (12)	0.12112 (12)	0.0283 (4)	
N1	−0.2590 (2)	0.44637 (12)	0.08106 (11)	0.0274 (4)	
N2	−0.5250 (2)	0.27281 (12)	−0.21818 (12)	0.0307 (4)	
C1	−0.5450 (3)	0.32180 (18)	−0.30356 (15)	0.0377 (5)	
H1A	−0.5970	0.3841	−0.2937	0.056*	
H1B	−0.6182	0.2841	−0.3430	0.056*	
H1C	−0.4334	0.3300	−0.3311	0.056*	
C3	−0.4545 (2)	0.31656 (14)	−0.14777 (14)	0.0283 (4)	
C12	−0.0597 (3)	0.65139 (15)	0.18233 (14)	0.0292 (4)	
H12	−0.0224	0.6698	0.2400	0.035*	
C13	−0.0398 (2)	0.71517 (14)	0.10920 (14)	0.0264 (4)	
C14	−0.0987 (3)	0.68593 (14)	0.02425 (14)	0.0277 (4)	
H14	−0.0889	0.7278	−0.0250	0.033*	
C9	−0.1702 (2)	0.59759 (14)	0.01219 (14)	0.0258 (4)	
C6	−0.3185 (2)	0.40896 (13)	0.00539 (14)	0.0257 (4)	
C10	−0.1897 (2)	0.53309 (14)	0.08529 (13)	0.0252 (4)	
C7	−0.3180 (2)	0.45347 (14)	−0.08064 (14)	0.0258 (4)	
C4	−0.4539 (3)	0.27152 (15)	−0.06247 (14)	0.0297 (4)	
H4	−0.4992	0.2094	−0.0565	0.036*	
C8	−0.3812 (3)	0.40837 (14)	−0.15486 (14)	0.0292 (4)	
H8	−0.3760	0.4387	−0.2112	0.035*	
C16	0.0901 (3)	0.83218 (16)	0.20952 (15)	0.0334 (5)	
H16A	−0.0092	0.8481	0.2457	0.050*	
H16B	0.1625	0.8883	0.2033	0.050*	
H16C	0.1546	0.7813	0.2386	0.050*	
C11	−0.1314 (3)	0.56496 (15)	0.17006 (14)	0.0302 (4)	
H11	−0.1433	0.5241	0.2199	0.036*	
C15	0.0592 (3)	0.86560 (15)	0.04613 (15)	0.0338 (5)	
H15A	0.1302	0.8348	0.0012	0.051*	
H15B	0.1157	0.9233	0.0674	0.051*	
H15C	−0.0511	0.8821	0.0197	0.051*	
C5	−0.3894 (3)	0.31642 (15)	0.00972 (15)	0.0305 (5)	
H5	−0.3916	0.2848	0.0655	0.037*	
C2	−0.5894 (3)	0.17565 (15)	−0.21258 (16)	0.0358 (5)	
H2A	−0.6818	0.1729	−0.1692	0.054*	
H2B	−0.4974	0.1332	−0.1937	0.054*	
H2C	−0.6320	0.1557	−0.2711	0.054*	

### 3,7-Bis(dimethylamino)phenothiazinium bisulfite (II) Atomic displacement parameters (Å^2^)

**Table:**

	*U*^11^	*U*^22^	*U*^33^	*U*^12^	*U*^13^	*U*^23^
S2	0.0290 (3)	0.0236 (3)	0.0298 (3)	0.00176 (19)	0.00111 (19)	0.00149 (19)
S1	0.0359 (3)	0.0239 (3)	0.0287 (3)	−0.0047 (2)	−0.0019 (2)	0.00329 (19)
O1	0.0306 (8)	0.0360 (9)	0.0406 (9)	0.0030 (6)	−0.0003 (6)	0.0104 (7)
O3	0.0353 (8)	0.0347 (8)	0.0308 (8)	−0.0022 (6)	−0.0045 (6)	0.0052 (6)
O2	0.0410 (8)	0.0280 (8)	0.0386 (9)	0.0057 (6)	0.0051 (7)	−0.0011 (6)
O4	0.0428 (9)	0.0264 (8)	0.0355 (8)	−0.0013 (6)	0.0023 (7)	0.0018 (6)
N3	0.0303 (9)	0.0246 (8)	0.0301 (9)	−0.0035 (7)	−0.0012 (7)	0.0011 (7)
N1	0.0296 (9)	0.0224 (8)	0.0301 (9)	0.0007 (7)	0.0009 (7)	0.0010 (7)
N2	0.0331 (9)	0.0261 (9)	0.0328 (9)	0.0002 (7)	−0.0021 (7)	−0.0030 (7)
C1	0.0370 (12)	0.0421 (13)	0.0339 (12)	−0.0052 (10)	−0.0004 (9)	−0.0014 (10)
C3	0.0243 (9)	0.0258 (10)	0.0347 (11)	0.0049 (8)	0.0002 (8)	−0.0036 (8)
C12	0.0308 (10)	0.0264 (10)	0.0306 (10)	−0.0002 (8)	−0.0006 (8)	−0.0008 (8)
C13	0.0233 (9)	0.0219 (9)	0.0340 (11)	0.0015 (7)	0.0019 (8)	−0.0003 (8)
C14	0.0279 (10)	0.0242 (10)	0.0308 (11)	−0.0012 (8)	0.0006 (8)	0.0020 (8)
C9	0.0228 (9)	0.0251 (10)	0.0296 (10)	0.0027 (7)	0.0016 (8)	0.0006 (8)
C6	0.0242 (9)	0.0217 (10)	0.0311 (10)	0.0028 (7)	0.0018 (8)	−0.0005 (8)
C10	0.0236 (9)	0.0205 (9)	0.0313 (10)	0.0012 (7)	−0.0001 (8)	0.0002 (8)
C7	0.0227 (9)	0.0221 (9)	0.0328 (10)	0.0014 (7)	0.0024 (8)	−0.0003 (8)
C4	0.0282 (10)	0.0228 (10)	0.0380 (11)	−0.0008 (8)	0.0006 (8)	−0.0001 (8)
C8	0.0304 (10)	0.0249 (10)	0.0321 (11)	0.0016 (8)	−0.0009 (8)	0.0015 (8)
C16	0.0389 (12)	0.0275 (10)	0.0339 (11)	−0.0070 (9)	−0.0026 (9)	−0.0009 (9)
C11	0.0357 (11)	0.0250 (10)	0.0299 (11)	−0.0002 (8)	−0.0008 (8)	0.0035 (8)
C15	0.0373 (11)	0.0268 (11)	0.0372 (12)	−0.0083 (9)	−0.0037 (9)	0.0061 (9)
C5	0.0333 (11)	0.0249 (10)	0.0331 (11)	0.0003 (8)	0.0020 (9)	0.0038 (8)
C2	0.0385 (12)	0.0258 (10)	0.0431 (13)	−0.0009 (9)	−0.0048 (10)	−0.0066 (9)

### 3,7-Bis(dimethylamino)phenothiazinium bisulfite (II) Geometric parameters (Å, º)

**Table:**

S2—O1	1.575 (3)	C13—C14	1.417 (3)
S2—O3	1.4680 (18)	C14—H14	0.9500
S2—O2	1.4385 (17)	C14—C9	1.378 (3)
S2—O4	1.4407 (18)	C9—C10	1.435 (3)
S1—C9	1.727 (2)	C6—C7	1.437 (3)
S1—C7	1.732 (2)	C6—C5	1.421 (3)
O1—H1	0.8400	C10—C11	1.424 (3)
N3—C13	1.345 (3)	C7—C8	1.374 (3)
N3—C16	1.468 (3)	C4—H4	0.9500
N3—C15	1.469 (3)	C4—C5	1.352 (3)
N1—C6	1.336 (3)	C8—H8	0.9500
N1—C10	1.340 (3)	C16—H16A	0.9800
N2—C1	1.465 (3)	C16—H16B	0.9800
N2—C3	1.342 (3)	C16—H16C	0.9800
N2—C2	1.463 (3)	C11—H11	0.9500
C1—H1A	0.9800	C15—H15A	0.9800
C1—H1B	0.9800	C15—H15B	0.9800
C1—H1C	0.9800	C15—H15C	0.9800
C3—C4	1.430 (3)	C5—H5	0.9500
C3—C8	1.421 (3)	C2—H2A	0.9800
C12—H12	0.9500	C2—H2B	0.9800
C12—C13	1.429 (3)	C2—H2C	0.9800
C12—C11	1.355 (3)		
			
O3—S2—O1	105.77 (9)	C5—C6—C7	116.46 (18)
O2—S2—O1	107.42 (10)	N1—C10—C9	125.96 (19)
O2—S2—O3	112.41 (10)	N1—C10—C11	117.33 (18)
O2—S2—O4	114.18 (10)	C11—C10—C9	116.71 (18)
O4—S2—O1	103.89 (9)	C6—C7—S1	120.50 (15)
O4—S2—O3	112.31 (9)	C8—C7—S1	117.84 (16)
C9—S1—C7	103.79 (10)	C8—C7—C6	121.66 (19)
S2—O1—H1	109.5	C3—C4—H4	119.7
C13—N3—C16	121.37 (17)	C5—C4—C3	120.7 (2)
C13—N3—C15	121.20 (17)	C5—C4—H4	119.7
C16—N3—C15	117.43 (17)	C3—C8—H8	119.8
C6—N1—C10	122.76 (18)	C7—C8—C3	120.37 (19)
C3—N2—C1	121.04 (19)	C7—C8—H8	119.8
C3—N2—C2	121.77 (19)	N3—C16—H16A	109.5
C2—N2—C1	117.17 (18)	N3—C16—H16B	109.5
N2—C1—H1A	109.5	N3—C16—H16C	109.5
N2—C1—H1B	109.5	H16A—C16—H16B	109.5
N2—C1—H1C	109.5	H16A—C16—H16C	109.5
H1A—C1—H1B	109.5	H16B—C16—H16C	109.5
H1A—C1—H1C	109.5	C12—C11—C10	122.5 (2)
H1B—C1—H1C	109.5	C12—C11—H11	118.8
N2—C3—C4	120.1 (2)	C10—C11—H11	118.8
N2—C3—C8	121.7 (2)	N3—C15—H15A	109.5
C8—C3—C4	118.19 (19)	N3—C15—H15B	109.5
C13—C12—H12	119.7	N3—C15—H15C	109.5
C11—C12—H12	119.7	H15A—C15—H15B	109.5
C11—C12—C13	120.6 (2)	H15A—C15—H15C	109.5
N3—C13—C12	120.60 (19)	H15B—C15—H15C	109.5
N3—C13—C14	121.19 (18)	C6—C5—H5	118.7
C14—C13—C12	118.21 (19)	C4—C5—C6	122.6 (2)
C13—C14—H14	119.6	C4—C5—H5	118.7
C9—C14—C13	120.83 (19)	N2—C2—H2A	109.5
C9—C14—H14	119.6	N2—C2—H2B	109.5
C14—C9—S1	118.04 (16)	N2—C2—H2C	109.5
C14—C9—C10	121.17 (19)	H2A—C2—H2B	109.5
C10—C9—S1	120.79 (16)	H2A—C2—H2C	109.5
N1—C6—C7	126.19 (18)	H2B—C2—H2C	109.5
N1—C6—C5	117.35 (19)		
			
S1—C9—C10—N1	−0.8 (3)	C9—C10—C11—C12	−0.5 (3)
S1—C9—C10—C11	179.98 (15)	C6—N1—C10—C9	0.6 (3)
S1—C7—C8—C3	−178.17 (15)	C6—N1—C10—C11	179.83 (18)
N3—C13—C14—C9	178.51 (19)	C6—C7—C8—C3	2.0 (3)
N1—C6—C7—S1	−0.4 (3)	C10—N1—C6—C7	0.1 (3)
N1—C6—C7—C8	179.4 (2)	C10—N1—C6—C5	−179.86 (18)
N1—C6—C5—C4	179.82 (19)	C7—S1—C9—C14	−179.56 (16)
N1—C10—C11—C12	−179.78 (19)	C7—S1—C9—C10	0.33 (18)
N2—C3—C4—C5	−177.30 (19)	C7—C6—C5—C4	−0.1 (3)
N2—C3—C8—C7	176.55 (19)	C4—C3—C8—C7	−2.5 (3)
C1—N2—C3—C4	172.77 (18)	C8—C3—C4—C5	1.8 (3)
C1—N2—C3—C8	−6.3 (3)	C16—N3—C13—C12	−2.1 (3)
C3—C4—C5—C6	−0.5 (3)	C16—N3—C13—C14	178.11 (18)
C12—C13—C14—C9	−1.3 (3)	C11—C12—C13—N3	−179.08 (19)
C13—C12—C11—C10	0.1 (3)	C11—C12—C13—C14	0.7 (3)
C13—C14—C9—S1	−179.09 (15)	C15—N3—C13—C12	178.25 (18)
C13—C14—C9—C10	1.0 (3)	C15—N3—C13—C14	−1.6 (3)
C14—C9—C10—N1	179.14 (19)	C5—C6—C7—S1	179.52 (15)
C14—C9—C10—C11	−0.1 (3)	C5—C6—C7—C8	−0.6 (3)
C9—S1—C7—C6	0.18 (18)	C2—N2—C3—C4	−5.5 (3)
C9—S1—C7—C8	−179.67 (16)	C2—N2—C3—C8	175.48 (19)

### 3,7-Bis(dimethylamino)phenothiazinium bisulfite (II) Hydrogen-bond geometry (Å, º)

**Table:**

*D*—H···*A*	*D*—H	H···*A*	*D*···*A*	*D*—H···*A*
O1—H1···O3^i^	0.84	1.77	2.609 (4)	175
C1—H1*C*···O4^ii^	0.98	2.39	3.349 (5)	167
C2—H2*B*···O2^iii^	0.98	2.56	3.506 (5)	163
C4—H4···O3^iv^	0.95	2.54	3.451 (5)	162
C12—H12···O4	0.95	2.46	3.372 (5)	162
C15—H15*B*···O2^v^	0.98	2.47	3.382 (5)	155
C15—H15*C*···O3^vi^	0.98	2.36	3.309 (5)	164
C16—H16*B*···O2^v^	0.98	2.32	3.283 (5)	167
C16—H16*C*···O4	0.98	2.52	3.326 (5)	139

Symmetry codes: (i) −*x*, −*y*+1, −*z*+1; (ii) −*x*, −*y*+1, −*z*; (iii) *x*−1/2, −*y*+1/2, *z*−1/2; (iv) −*x*−1/2, *y*−1/2, −*z*+1/2; (v) −*x*+1/2, *y*+1/2, −*z*+1/2; (vi) *x*−1/2, −*y*+3/2, *z*−1/2.
