# Supplementary material for: Hydrogen bonds and π–π inter­actions in two new crystalline phases of methyl­ene blue
Source: Acta Crystallogr E Crystallogr Commun. 2018 Apr 17;74(Pt 5):587–93. doi: 10.1107/S2056989017017881 (PMC5947468; doi:10.1107/S2056989017017881)
Supplement: Supplementary file 5 [file e-74-00587-sup5.pdf]

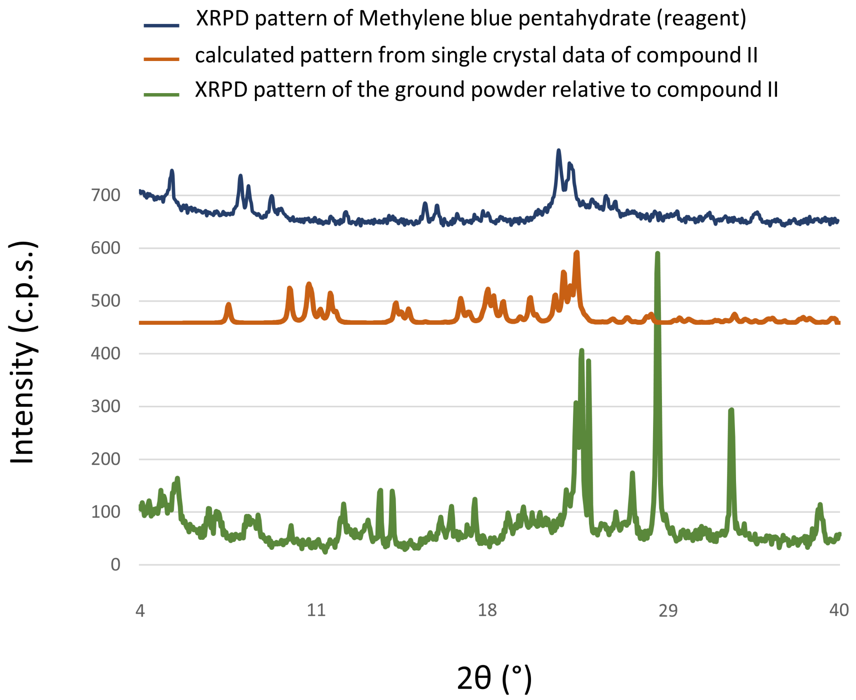

Figure S1. Stacked XRPD patterns of grinding product of $HgSO_4$ with MB pentahydrate (green), commercial MB pentahydrate (blue) and calculated profile for compound II calculated by the determined structure (orange).
